# Comparison of inequity in health-related quality of life among unemployed and employed individuals in China

**DOI:** 10.1186/s12889-020-10038-3

**Published:** 2021-01-06

**Authors:** Yaxin Zhao, Zhongliang Zhou, Xiaojing Fan, Rashed Nawaz, Dantong Zhao, Tiange Xu, Min Su, Dan Cao, Chi Shen, Sha Lai

**Affiliations:** 1 School of Public Health, Health Science Center, Xi’an Jiaotong University, No.76 West Yanta Road, Xi’an, 710061 Shaanxi China; 2 School of Public Policy and Administration, Xi’an Jiaotong University, No. 28 Xianning West Road, Xi’an, 710049 Shaanxi China; 3 School of Public Administration, Inner Mongolia University, No. 235 College Road, Hohhot, 010021 Inner Mongolia China

**Keywords:** Health-related quality of life, Health equity, EQ-5D, Unemployment, Coarsened exact matching, China

## Abstract

**Background:**

In China, achieving health equity has been regarded as a key issue for health reform and development in the current context. It is well known that unemployment has a negative effect on health. However, few studies have addressed the association between unemployment and inequity in health-related quality of life (HRQOL). This study aims to compare the inequality and inequity in HRQOL between the unemployed and employed in China.

**Methods:**

The material regarding this study was derived from the Chinese National Health Services Survey of Shaanxi Province for 2013. We controlled for confounding factors by utilizing the coarsened exact matching method. Finally, 7524 employed individuals and 283 unemployed individuals who were 15 to 64 years old in urban areas were included in this study. We used HRQOL as the outcome variable, which was evaluated by using the Chinese version of EQ-5D-3L. The health concentration index, decomposition analysis based on the Tobit model, and the horizontal inequity index were employed to compute the socioeconomic-related equity between the unemployed and employed and the contribution of various factors.

**Results:**

After matching, unemployed people tended to have poorer EQ-5D utility scores than employed people. There were statistically pro-rich inequalities in HRQOL among both employed and unemployed people, and the pro-rich health inequity of unemployed people was substantially higher than that of employed people. Economic status, age, education, smoking and health insurance were the factors influencing inequality in HRQOL between employed and unemployed individuals. Education status and basic health insurance have reduced the pro-rich inequity in HRQOL for unemployed people.

**Conclusion:**

It is suggested that unemployment intensifies inequality and inequity in HRQOL. According to policymakers, basic health insurance is still a critical health policy for improving health equity for the unemployed. Intervention initiatives aiming to tackle long-term unemployment through active labour market programmes, narrow economic gaps, improve educational equity and promote the health status of the unemployed should be considered by the government to achieve health equity.

## Background

Health equity has gradually become a research hotspot in the field of health system reform [1, 2]. Achieving health equity has been a source of concern with a strong degree of support and response from all countries of the world [3]. China also regards the realization of health equity as the key issue of health reform and development in the current context. Specifically, the planning outline of “Healthy China 2030” has proposed that we should focus on the health problems of vulnerable groups of people to achieve health equity [4]. As an important economic and material basis for people, economic status is an important factor affecting health and health inequity. The widening of the income gap in China has also aroused widespread public concern. Empirical studies about health inequality have commonly used economic status to analyse health inequalities and inequities [5–7]. As Wagstaff suggested, in order to analyse socioeconomic health inequities, health-related information must be supplemented by data on socioeconomic status. There are many approaches to measuring socioeconomic status such as income, expenditure, or consumption [8]. Health inequalities are not only affected by physiological conditions but also widely determined by socioeconomic characteristics and inequalities may be further widened by unemployment [9]. The World Health Organization proposed that each country should set up health equity monitoring systems to reduce health inequalities by collecting data on key indicators such as employment status, which can be determined by the labour market [10]. Unlike retired people, most unemployed people quit the labour force for non-physiological reasons and cannot sell their labour at a balanced price in the market [11]. In addition, some articles have examined the impact of unemployment and income inequalities on the degree of criminality and mental health [12], as well as the associations between unemployment, income inequality and suicide mortality [13]. It is of great practical significance to compare and measure the income-related health inequality between unemployed and employed individuals in China.

There is a body of literature that has explored the association between unemployment and lifestyle behaviours (e.g., alcohol consumption and smoking) [14, 15], the effects of unemployment on mental health (e.g., depression, mental disorder and suicide thoughts) [16–19], and the effects of unemployment on physical health outcomes (e.g., mortality) and subjective health outcomes (e.g., self-reported health) [20–22]. Empirical evidence has demonstrated that unemployment has a severely negative effect on health and that unemployment also significantly raises the risk of mental disorders and suicide [23, 24]. In addition, some international studies have revealed that unemployed people were significantly more likely to have poor self-reported health than employed people [20–22]. Unemployment may lead households into a cycle of poverty [25] and households may be disadvantaged in terms of health and access to health care services, which leads to changes in health equity. Therefore, it is logical to start from the key groups and to carry out research on inequity in health-related quality of life among the unemployed. It is highly important to prevent unemployed individuals from falling into long-term health problems and poverty, to improve the precision of poverty alleviation policies and to promote the construction of “Healthy China 2030”.

Despite many health indicators being used to assess the effect of unemployment on health, health-related quality of life remained remarkably absent from health measurement [26]. Health-related quality of life (HRQOL) is generally considered a key measurement indicator of health care outcomes and is constructed multidimensionally in relation to a person’s self-perceived health [27]. The EuroQol 5 dimensions (EQ-5D) is a standardized instrument, and is most commonly used for measuring the quality of life in public health research [28, 29]. Some recent studies have examined the correlates of unemployment and HRQOL by using the MOS 8-item short-form health survey instrument, SF-12 instrument and SF-36 instrument [30–33], but studies that used the EQ-5D instrument to explore the relation of unemployment and HRQOL are relatively few in number. The EQ-5D instrument is easy to operate and has high applicability, as it has been tested in a large-sample and large-scale Chinese National Health Services Survey. Most importantly, the EQ-5D has time trade-off values based on a conversion of Chinese preferences for EQ-5D health states, which can more accurately reflect the HRQOL of Chinese residents [34].

Despite the importance of unemployment in models of social and ecological determinants of health, we know very little about the relationship of unemployment and health inequities in HRQOL. Leaving unemployment and employment out of public health inequity research creates a blind spot. This paper thus contributes to two strands of literature on the empirical evaluation of HRQOL for unemployed individuals and health inequities in China. First, the existing literature on the relation of unemployment and health has focused on mental health and self-assessed health [16–22], but the literature on the association of unemployment and HRQOL is scarce. In addition, the inequity in HRQOL for the unemployed and employed has not yet been evaluated using the CI and HI. Second, with the instruments for measuring quality of life, studies attempt to investigate the relationship between unemployment and HRQOL by using the EQ-5D instrument are very limited [30–32]. Third, in terms of methods, researchers often analysed HRQOL and inequities in HRQOL by using descriptive statistical analysis and linear regression, lacking a scientific method to balance the comparison groups; thus, such approaches cannot reflect the ceiling effect of EQ-5D and measure the inequity of HRQOL quantitatively [33]. In this article, we make an initial contribution to filling what is a rather large gap in the public health inequity research by investigating the relationship between unemployment and health inequities in HRQOL.

Based on the abovementioned background, we have attempted to answer three main questions: (1) What is the health utility of the employed and the unemployed in China? Is the health utility value of the unemployed higher than that of the employed? (2) What are the levels of inequality and inequity in HRQOL between the employed and unemployed? Are the concentration index and horizontal equity index of the unemployed higher than those of the employed? (3) How do relevant factors contribute to the health inequalities in HRQOL between the employed and unemployed? In this paper, we have calculated and compared the health utility between the employed and unemployed in China. In addition, we decomposed the inequality and analysed the inequity in HRQOL between the employed and unemployed in China. Careful consideration of unemployment in public health research can allow us to make better progress towards achieving health equity.

## Methods

### Data and Sample

This study draws upon data from the Chinese National Health Services Survey of Shaanxi Province in 2013, a representative cross-sectional survey of households and individuals (adults and children) launched in 1993 by the National Health Commission of China every 5 years. The 5th wave survey adopted a multi-stage stratified cluster sampling method that was conducted in Shaanxi Province. In the first stage, this survey selected 32 counties (districts); 160 towns (streets) were selected in the next stage, and 320 villages (communities) were selected in the final stage. Finally, 20,700 households (57,529 people) were identified [34, 35]. With this survey, we attached great importance to data quality and implemented a considerable number of quality control measures in the following stages: survey design, training of investigators, field investigation and data collection. Based on a series of quality control measures, high response rates (> 85%) were achieved for this survey [36].

The Chinese National Health Services survey also focused on the health status and the health services need and utilization of the Chinese residents, covering a broad range of information on socioeconomic characteristics (e.g., age, gender, education status and economic level), health (e.g., self-assessed health and HRQOL) and health service utilization. In this study, 10,337 employed and 285 unemployed respondents whose ages ranged from 15 to 64 years in urban areas were identified in the final sample before matching.

### Variables and Measures

#### Health-related quality of life variables

We used EQ-5D health utility as the outcome variable. HRQOL was measured by the classic 3-level EQ-5D (EQ-5D-3L), which has been widely validated and utilized worldwide [37]. The EQ-5D is a self-report questionnaire, that includes five dimensions: (1) mobility, (2) self-care, (3) usual activities (such as work, studies, housework and leisure activities), (4) pain/ discomfort, and (5) anxiety/depression. The three response alternatives to the five dimensions mentioned above are (1) no problem, (2) some problems, and (3) extreme problems [38]. Finally, we used the conversion for Chinese preferences to generate the score of EQ-5D utility between the unemployed and employed, which ranges from − 0.1490 (stands for the worst health) to 1 (stands for the full health) [39]. By combining one level of each of the five dimensions, a total of 243 possible health states can be defined, and a score for EQ-5D utility for all 243 health states can be calculated based on the results in additional files in Table A.

#### Control variables

In light of the existing literature, we controlled for variables including socio-demographic characteristics and health behaviour related to inhabitants, such as gender (0 = male, 1 = female), age (in years), per capita annual income (Yuan) (1 = lowest group, 0 = other; 1 = lower group, 0 = other; 1 = medium group, 0 = other; 1 = higher group, 0 = other; 1 = highest group, 0 = other), marital status (1 = single, 0 = other; 1 = marriage, 0 = other; 1 = widowed and divorced, 0 = other), education status (1 = elementary school and below, 0 = other; 1 = middle school, 0 = other; 1 = senior high school, 0 = other; 1 = college degree and above, 0 = other), health insurance (1 = no, 0 = other; 1 = basic medical insurance, 0 = other; 1 = commercial insurance and other insurance, 0 = other;), smoking status (1 = no smoking, 0 = other; 1 = non-daily smoking, 0 = other; 1 = daily smoking, 0 = other;) and drinking status (0 = no drinking, 1 = drinking).

### Statistical analysis

#### Coarsened exact matching

A rough comparison of equity in HRQOL between the unemployed and employed would ignore the fact that there may be other potential confounding factors. Therefore, in this article, we adopted the coarsened exact matching method, which is a new technique for improving the assessment of causal inference between two groups by controlling for potentially confounding variables [40, 41]. The purpose of this method is to keep the distribution of covariates between the treatment group and the control group as balanced as possible, thereby improving the comparability between the two groups. The exact matching algorithm was used to accurately match the research objects in each layer according to the empirical distribution of samples. During the matching process, weighted variables were generated to ensure that there were at least one treatment group and one control group in each layer; otherwise, the research objects were deleted. Finally, the matched research objects were retained, and the matched data were employed for the analysis [5]. The multivariate imbalance measure *L*_1_ was employed to ensure the balance before and after matching. *L*_1_ ranges from 0 to 1, where 1 indicates that the data of two comparison groups are completely unbalanced and a smaller value indicates a better balance between comparison groups. The multivariate imbalance was measured by Eq.1 [41]:

1 $$ {L}_1\left(f,g;H\right)=\frac{1}{2}\sum \limits_{\varepsilon_1\dots {\varepsilon}_K\in H(X)}\left|{f}_{\varepsilon_1\dots {\varepsilon}_k}-{g}_{\varepsilon_1\cdots {\varepsilon}_k}\right| $$

*f* and *g* are the relative frequencies for the distributions of the two groups. *H*(*X*) represents the Cartesian product of *H*(*X*_1_) × ⋯ × *H*(*X*_*k*_). ${f}_{\varepsilon_1\dots {\varepsilon}_k}$ indicates the relative frequency for samples falling into the cell with coordinates *ε*_1_…*ε*_*k*_ of the multivariate cross-tabulated of the treated units and ${g}_{\varepsilon_1\dots {\varepsilon}_k}$ for the control units.

### Analysis of inequity in health-related quality of life

#### Concentration index

Health inequality and health inequity appear to be similar, but there is a difference between them. Health inequality is a relative condition that refers to differences in health status or in the distribution of health determinants among different population groups [42]. However, strict equality in health for all would not be a feasible or achievable goal because some determinants of health are unavoidable and are beyond human control [8]. We measured health inequality with the concentration index (CI). CI has been widely accepted as a standard method for measuring the socioeconomic-related inequality of health status [6]. The CI value is between − 1 and 1. A positive CI indicates that health is more concentrated among members with higher per capita household income; by contrast, 0 indicates that there is no inequality [43]. The concentration index was computed with Eq.2:

2 $$ C=2\mathit{\operatorname{cov}}\left(x,h\right)/\mu $$

where *C* denotes the concentration index, *x* refers to HRQOL, *μ* is the average of EQ-5D utility value, and *h* symbolizes the ranking of per capita household income.

#### Decomposition of the concentration index

The decomposition analysis is intended to decompose the concentration index into the contribution of every variable to the inequality in HRQOL. Empirical research has demonstrated that the work, age, education, lifestyle behaviours, health insurance, economic level and resources are the immediate causes of some of these health inequities [44]. Therefore, we selected the contributing variables that include age, gender, education, income, marriage, health behaviours (smoking and drinking) and health insurance to decompose the inequality in HRQOL. These variables were divided into need variables and non-need variables of HRQOL to calculate the horizontal inequity index. Need variables, or *x*_,_ referred to the unavoidable determinants of health, while the control, or *z*, non-need variables referred to the avoidable determinants of health. As Wagstaff and a number of studies have suggested, age and gender are commonly used to reflect unavoidable determinants of health in the analysis of health inequity [5, 42, 45]. In addition, the EQ-5D utility value generally has a ceiling effect, with only 3 levels in each dimension; that is, most respondents are in good physical condition, and most health utility values calculated from it are 1. If the effect were ignored in the analysis of health-related quality of life factors, the traditional regression method would inevitably produce false estimates. Therefore, to consider the limited dependent variable, we used the Tobit model to solve the problem. The decomposition analysis based on the Tobit model [42] was the commonly used method shown in Eq.3:

3 $$ {y}_i=\alpha +\sum \limits_j{\beta}_j^m{x}_{ji}+\sum \limits_k{\gamma}_k^n{z}_{ki}+{\varepsilon}_i $$

where *y*_*i*_ is the score for EQ-5D utility; *x* are the need variables of HRQOL (e.g., gender and age); *z* indicates the non-need variables of HRQOL (e.g., health insurance, education status, marital status, economic level and health behaviour); ${\beta}_j^m$ and ${\gamma}_k^n$ indicate the marginal effects (dy/dx) of every variable; and *ε*_*i*_ refers to the error term. The decomposition of the concentration index *C* can be written as follows:

4 $$ C=\sum \limits_j\left({\beta}_j^m{\overline{x}}_j/\mu \right){C}_j+{GC}_{\varepsilon }/\mu $$

where *μ* represents the mean of EQ-5D utility, *C*_*j*_ denotes the concentration index of *x*_*j*_, and ${\overline{x}}_j$ is the mean for *x*_*j*_. The last term is the concentration index of *ε*.

#### Horizontal inequity index

Inequity implies a state that results from a lack of fairness, which is related to a normative view of social justice and is the most relevant to our discussion about the pursuit of health equity [7]. Health inequities are avoidable, unfair and potentially remediable inequalities in health between groups of people [42]. Most of the literature on health economics has employed horizontal equity as the criterion of equity, stating that people with equal health needs should be treated equally [46]. Thus, we measured health inequity by using the horizontal inequity index (HI). The horizontal inequity (HI) of HRQOL indicates the inequality in HRQOL by eliminating the contribution of need variables. In the present investigation, the horizontal inequity index was generated by subtracting the contribution of the need variables (e.g., gender and age) from the concentration index of HRQOL [43]. The HI is positive if there exists a pro-rich inequity and vice versa.

## Results

### Matching results

Summary statistics for the employed and unemployed individuals before and after coarsened exact matching are presented in Table 1. The results before matching indicate that the differences in socio-demographic characteristics among the two groups were statistically significant except for drinking alcohol and smoking status. Specifically, results after matching demonstrated that the differences in socio-demographic characteristics between employed and unemployed individuals were statistically insignificant, except for medical health insurance, which was controlled in the health inequity analysis. Additionally, the results of the multivariate imbalance measure L_1_ are shown in additional files in Table B. The value of L_1_ (6.17*10–15) between employed and unemployed individuals after matching was obviously lower than that before matching (0.448), which signified that the matching effect was good and that the two groups became more comparable. As presented in Table 1, data for a total of 7857 residents were collected in this study, with data for 7574 employed and 283 unemployed residents after coarsened exact matching.

**Table 1:** Summary statistics and description of independent variables before and after coarsened exact matching

Variables	Description of variables		Before matching				After matching		
		Obs.	Employed	Unemployed	*p*-value	Obs.	Employed	Unemployed	*p*-value
N		10,622	10,337	285		7857	7574	283	
Gender		10,622				7857			
Male^a^			5472 (52.94)	134 (47.02)	0.048		3563 (47.35)	134 (47.35)	1.000
Female	Female = 1, male = 0		4865 (47.06)	151 (52.98)			3961 (52.65)	149 (52.65)	
Age (years)		10,622				7857			
15–29^a^			2055 (19.27)	27 (9.47)	< 0.001		718 (9.54)	27 (9.54)	1.000
30–44	30–44 = 1, other = 0		3974 (38.44)	102 (35.79)			2712 (36.04)	102 (36.04)	
> 45	> 45 = 1, other = 0		4308 (41.68)	156 (54.74)			4094 (54.42)	154 (54.42)	
Marital status		10,622				7857			
Single ^a^			1065 (10.30)	15 (5.26)	< 0.001		399 (5.30)	15 (5.30)	1.000
Marriage	Marriage = 1, other = 0		8984 (86.91)	247 (86.67)			6567 (87.28)	247 (87.28)	
Widowed and divorced	Widowed and divorced = 1, other = 0		288 (2.79)	23 (8.07)			558 (7.42)	21 (7.42)	
Education status		10,622				7857			
Elementary school and below ^a^			2028 (19.62)	20 (7.02)	< 0.001		532 (7.07)	20 (7.07)	1.000
Middle school	Middle school = 1, other = 0		4600 (44.50)	116 (40.70)			3084 (40.99)	116 (40.99)	
Senior high school	Senior high school = 1, other = 0		2143 (20.73)	126 (44.21)			3323 (44.17)	125 (44.17)	
College degree and above	College degree and above = 1, other = 0		1566 (15.15)	23 (8.07)			585 (7.77)	22 (7.77)	
Health insurance		10,622				7857			
No ^a^			227 (2.20)	11 (3.86)	< 0.001		143 (1.89)	11 (3.89)	< 0.001
Basic medical insurance	Basic medical insurance = 1, other = 0		7382 (71.43)	158 (55.44)			5504 (72.67)	158 (55.83)	
Commercial insurance and other insurance	Commercial insurance and other insurance = 1, other = 0		2726 (26.38)	116 (40.70)			1927 (25.44)	114 (40.28)	
Smoking status ^b^		10,610				7848			
No smoking ^a^			6801 (65.86)	189 (66.78)	0.262		5030 (65.68)	187 (66.55)	0.286
Non-daily smoking	Non-daily smoking = 1, other = 0		464 (4.49)	7 (2.47)			330 (4.28)	7 (2.49)	
Daily smoking	Daily smoking = 1, other = 0		3062 (29.65)	87 (30.74)			2207 (30.04)	87 (30.96)	
Drinking alcohol ^b^		10,444				7717			
No ^a^			7659 (75.35)	213 (76.34)	0.703		5718 (76.85)	211 (76.17)	0.792
Yes	Yes = 1, No = 0		2506 (24.65)	66 (23.66)			1722 (23.15)	66 (23.83)	
Economic status (Yuan) ^b^		10,322				7857			
Lowest group ^a^			2047 (19.83)	74 (25.96)	< 0.001		1954 (25.80)	73 (25.80)	0.998
Lower group	Lower group = 1, other = 0		2064 (20.00)	58 (20.35)			1526 (20.14)	57 (20.14)	
Medium group	Medium group = 1, other = 0		2056 (19.92)	65 (22.81)			1740(22.97)	65 (22.97)	
Higher group	Higher group = 1, other = 0		2074 (20.09)	48 (16.84)			1284 (16.96)	48 (16.96)	
Highest group	Highest group = 1, other = 0		2081 (20.16)	40 (14.04)			1070 (14.13)	40 (14.13)	

N (%) and chi-square test were performed for categorical variables and binary variables

^a^Reference group in the Tobit regression

^b^There were missing values. Before the matching, there were 12 missing values for smoking status, 178 missing values for drinking alcohol and 300 missing values for economic status. After the matching, there were 9 missing values for smoking status and 140 missing values for drinking alcohol

### Description of EQ-5D dimensions

The distribution of the three response alternatives for EQ-5D in each dimension for the employed and unemployed residents are shown in Table 2. After matching, the results indicated that the unemployed residents reported a higher proportion of some problems/extreme problems in five dimensions than the employed residents did, and this result was statistically significant. Table 3 presents the EQ-5D health utility scores with a conversion based on Chinese preferences between the employed and unemployed in China. After matching, the results indicated that the differences in the mean of EQ-5D utility scores and the utility scores for the five dimensions were statistically significant between the employed and unemployed people. Moreover, unemployed residents tended to exhibit significantly higher EQ-5D utility scores than employed residents. Thus, unemployed people are significantly expected to suffer from health troubles in each of the EQ-5D dimensions significantly more than employed people. We analyzed the data before matching in additional files in Table C and Table D and found that the results were almost consistent with the results after matching, which also indicated that the results were robust.

**Table 2:** Distribution of the three response alternatives for EQ-5D in each dimensions for the employed and unemployed

EQ-5D dimensions	Employed (*N* = 7574)			Unemployed (*N* = 283)			χ^2^	*P*-value
	No problem	Some problems	Extreme problems	No problem	Some problems	Extreme problems		
Mobility	97.87	2.01	0.12	93.29	6.01	0.71	27.499	< 0.0001
Self-care	99.22	0.70	0.08	95.76	3.53	0.71	37.801	< 0.0001
Activity	98.59	1.22	0.19	93.99	4.95	1.06	37.975	< 0.0001
Pain	93.51	6.21	0.28	86.57	12.72	0.71	21.017	< 0.0001
Anxiety	95.69	4.05	0.27	90.11	9.19	0.71	19.806	< 0.0001

**Table 3:** The values for EQ-5D utility and each dimension for the employed and unemployed

EQ-5D dimensions	Employed		Unemployed		*P*-value
	Mean	S.D.	Mean	S.D.	
Mobility	−0.0024	0.0166	−0.0077	0.0310	< 0.0001
Self-care	−0.0009	0.0105	−0.0052	0.0259	< 0.0001
Activity	−0.0013	0.0118	−0.0057	0.0252	< 0.0001
Pain	−0.0065	0.0256	−0.0134	0.0360	< 0.0001
Anxiety	−0.0042	0.0204	−0.0094	0.0299	< 0.0001
EQ-5D	0.9809	0.0736	0.9510	0.1358	< 0.0001

### Inequity in HRQOL between Employed and Unemployed People

The CIs for the EQ-5D utility scores between the employed and unemployed are presented in Table 4. The overall CIs for the EQ-5D utility values for both employed (0.0028) and unemployed (0.0089) individuals were positive, signifying that there is a statistically pro-rich inequality in HRQOL between employed and unemployed people in Shaanxi Province, China. This indicates that overall better HRQOL are more concentrated in two groups with higher economic levels. In contrast, the respondents with lower economic levels had more health issues than those with higher economic level residents. Furthermore, the degree of inequality in HRQOL among unemployed people was higher than that among employed people.

**Table 4:** Decomposition of the concentration index in HRQOL among the employed and unemployed

Variables	Employed			Unemployed		
	dy/dx	Contribution	%	dy/dx	Contribution	%
Female (Ref: Male)	0.0022	0.0001	1.8436	0.0375*	0.0010	11.0270
30–44 (Ref: 15–29)	− 0.0055	− 0.0001	−2.1701	− 0.0210	− 0.0002	−2.7018
> 45	− 0.0219***	0.0004	15.7767	− 0.0361	0.0007	7.6776
Marriage (Ref: Single)	−0.0011	0	−0.1188	− 0.0145	− 0.0001	−0.8602
Widowed and divorced	−0.0072***	0	0.2393	−0.0936*	0.0003	3.3535
Middle school (Ref: Elementary school and below)	0.0146***	−0.0007	−24.7037	0.1092***	−0.0056	−61.1708
Senior high school	0.00171***	0.0004	14.0376	0.1112***	0.0026	28.2023
College degree and above	0.0183***	0.0005	17.8967	0.0685	0.0019	20.8629
Basic health insurance (Ref: No health insurance)	0.0008	−0.0001	−2.4595	0.0202	−0.0011	−12.4034
Commercial insurance and other insurance	0.0020	0.0002	5.5833	0.0169	0.0006	8.9765
Non-daily smoking (Ref: No smoking)	−0.0048	0	0.3741	0.0169	0	−0.5590
Smoking daily	−0.0024	0	0.5066	0.0564**	0.0008	8.6028
Drinking alcohol (Ref: No drinking)	0.0067***	0.0001	3.8739	0.0268	0.0005	5.2067
Lower group (Ref: Lowest group)	0.0029	−0.0002	−7.4199	0.0465**	−0.0040	−44.0800
Medium group	0.0054**	0.0670	3.3306	0.0415*	−0.0006	−0.2416
Higher group	0.0045*	0.0003	12.1718	0.0270	0.0023	25.6588
Highest group	0.0058**	0.0008	30.2274	0.0426*	0.0071	78.7459
CI	0.0028			0.0089		

*p < 0.1, **p < 0.05, ***p < 0.01

**Fig. 1:**
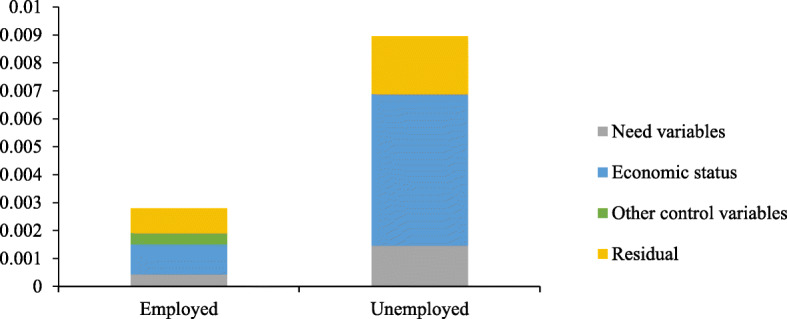
Classification analysis of inequity in HRQoL for the employed and the unemployed

The overall decomposition analysis for the EQ-5D utility values between the employed and unemployed are presented in Table 4. The marginal effect estimates from the two groups suggested that education status had a positive marginal effect, indicating that a higher level of education was significantly related to higher EQ-5D utility values. However, age had a negative marginal effect, suggesting that being older was associated with a decline in HRQOL. As distinguished in Table 4, the key contributions were from economic level (38.31%), age (13.61%) and educational status (7.23%) for the employed, whereas the three key contributors were economic status (60.08%), educational status (− 12.11%) and smoking status (8.04%) for the unemployed. Furthermore, the effects of different types of health insurances have different directions. The basic health insurance had a negative contribution and reduced the pro-rich impact on HRQOL for the employed and unemployed. However, the commercial insurance and other insurance had a positive contribution to the inequity of HRQOL. In addition, health behaviours also contributed to increasing pro-rich inequality in HRQOL. As depicted in Fig. 1, the contributors of need variables, economic status, other control variables and the residual to the inequality in HRQOL were above the level of the horizontal equity line, implying that these variables increased the pro-rich inequity between the employed and unemployed.

The horizontal inequity index of HRQOL is also presented in Table 5. After deduction of the contributions of the need variables in health (e.g., age and gender) from the concentration index of EQ-5D utility value, the horizontal inequity indexes of the HRQOL between employed and unemployed individuals were 0.0024 and 0.0075, respectively, which indicated a pro-rich inequity in HRQOL between the unemployed and employed. In addition, the horizontal inequity was greater for unemployed individuals than for employed individuals. To test the robustness of the results, we analysed the inequity of EQ-5D scores for the employed and unemployed before matching in additional files in Table E and Table F. The horizontal inequity indexes of the HRQOL among employed and unemployed individuals before matching were the same as the results after matching, which indicated that the results are robust.

**Table 5:** Horizontal inequity of EQ-5D scores for the employed and unemployed

	Employed	Unemployed
Contribution of need variables (age-gender)	0.0004	0.0014
Contribution of control variables	0.0015	0.0055
Residual	0.0009	0.0020
CI	0.0028	0.0089
HI	0.0024	0.0075

## Discussion

In the present research, we have assessed the long-studied topic of HRQOL in the research area of health care and economics. Based on the matched data, our results demonstrated that unemployed people reported lower HRQOL than employed people. In addition, unemployed people had higher levels of pro-rich inequality and horizontal inequity in HRQOL, which was mainly related to factors of economic status, educational status, age, smoking and health insurance. Therefore, there are three aspects of this study that should be discussed.

First, the most fascinating finding was that there was statistically higher EQ-5D utility for employed individuals compared with unemployed individuals, and this study was the first to assess HRQOL among the employed and unemployed individuals by using the EQ-5D-3L instrument in China. This indicated that unemployment was associated with poor HRQOL. This result is consistent with several reports that unemployed people are likely to have poorer HRQOL than employed people [26, 31, 47]. Specifically, this may be because people who experience unemployment are deprived of these benefits (e.g., income, social contact, status and activity), face greater financial and mental stress, and have lower health care utilization.

Second, the present study verified that the CI of HRQOL between the employed and the unemployed were both positive values, suggesting that the higher HRQOL was concentrated among rich men between the employed and unemployed people in Shaanxi. Additionally, the CI of the EQ-5D utility values among the unemployed was higher than that among the employed, which suggested that the unemployed had a higher pro-rich inequality in HRQOL than the employed. This study fills the gap in the literature by the comparing socioeconomic-related inequality between employed and unemployed individuals. Since previous research has not primarily focused on health inequality between employed and unemployed people in China, we can only compare this estimation with previous research on different kinds of people. Consistent with several previous reports of the different insured populations [5], findings from the marginal effect estimates among employed and unemployed individuals indicated that an advanced level of education was connected to better HRQOL. This might be because highly educated people have a stronger health awareness and better ability to cope with diseases. Moreover, as expected, age had a negative marginal effect, signifying that elderly people tend to have lower health outcomes. Furthermore, our findings indicate that the economic level intensified the pro-rich inequality in HRQOL and that the gap between the rich and poor people remains the key factor influencing inequality in HRQOL between the employed and unemployed, which was in agreement with previous studies of the different populations [5, 34, 42]. Apart from the economic level, age, educational status, health insurance and health behaviour also contributed to inequality in HRQOL. From the government point of view, this research demonstrated that basic health insurance schemes and educational level would reduce the pro-rich inequity in HRQOL for unemployed people. Ensuring basic medical insurance and enhancing education remain important health policies to reduce the inequity in HRQOL [33]. In contrast, commercial insurance and other insurance also increased the pro-rich inequity of HRQOL in unemployed individuals. It seems that commercial insurance has focused on efficiency due to market competition and most of the beneficiaries have been high-income groups. Smoking and drinking also contributed to increasing the pro-rich inequity of HRQOL, which is consistent with several reports that tobacco use and alcohol consumption were adverse health consequences and significant causes of health inequity worldwide [48]. This is because those who experienced social disadvantage, with low incomes or unemployment, were more likely to become regular smokers [49]. The purchase of tobacco products by households of tobacco users with lower socioeconomic status exacerbates poverty and social inequities by reducing the funds available for basic expenditures such as housing, clothing and food [50].

Third, our results regarding the inequity in HRQOL may be attractive to policy makers in regions where unemployment has increased significantly due to the financial crisis. In our research, after subtracting the contribution of the need variables, we found that the horizontal inequity index illustrated not only that there was pro-rich inequity in HRQOL between the two groups but also that this inequity for unemployed individuals was still higher than that for employed individuals, which may be explained by the reduction in income associated with unemployment [25, 33]. People have unequal access to social resources, including health resource, resulting in an increase in horizontal inequity in HRQOL. Specifically, unemployment had a negative effect on health equity and increased the pro-rich inequity in HRQOL. Therefore, when promoting a “Healthy China 2030” to achieve health equity among different groups, such as the unemployed and employed groups, the government should consider the contribution of education and basic health insurance schemes to reduce pro-rich inequity.

### Strengths and limitations

The current investigation has three key strengths. First, it is the first to compare the HRQOL of unemployed and employed individuals by using the EQ-5D-3L based on a conversion for Chinese preferences. Furthermore, we offer well-informed estimates of the associations between unemployment and socioeconomic-related inequality and inequity in Chinese HRQOL. The third key strength is that the findings of this investigation were based on a stronger balance between the unemployed and the employed groups by using the coarsened exact matching method.

At the same time, we acknowledge that the present study also has some limitations. First, in the data material, self-reported information regarding socioeconomic variables and EQ-5D scores may contain measurement errors and possibly introduce recall bias. Second, the data derived from Shaanxi Province and our conclusion may not be generalizable to all of China. Third, we must indicate that without valid instrumental variables, causal interpretations are hazardous, and possible endogenous problems could not be omitted in these cross-sectional data. Therefore, we refer to associations between unemployment and HRQOL. Fourth, the present study was subject to possibly unobserved confounding factors, such as disability status, access to healthy food, and social interaction. Finally, in the analytical techniques, the coarsened exact matching may exclude some observations that are very dissimilar in observable characteristics to obtain two groups that are as similar as possible.

## Conclusions

In conclusion, the unemployed had poorer HRQOL than the employed in this study in China, and the unemployed had higher pro-rich inequity in HRQOL than the employed. Unemployment is linked with health-related quality of life and inequality in HRQOL. It appeared that unemployment intensified the inequality and inequity in HRQOL. The major contributors to inequality in HRQOL were economic status, education status, age, smoking and health insurance for employed and unemployed residents. Education status and basic health insurance have positive effects on decreasing inequity in HRQOL among the unemployed. Intervention initiatives aiming to tackle long-term unemployment through active labour market programmes, narrow economic gaps, improve educational equity and improve the health status of the unemployed should be considered by the government to achieve greater health equity. Additionally, the socialization of health insurance for the unemployed should be improved.

## Supplementary Information

**Additional file 1: Table A.** Chinese time trade-off utility values for EQ-5D health states. **Table B.** The multivariate imbalance measure L_1_ before and after coarsened exact matching. **Table C.** Distribution of the three response alternatives for EQ-5D in each dimension for the employed and unemployed before matching. **Table D.** The values for EQ-5D utility and each dimension for the employed and unemployed before matching. **Table E.** Decomposition of the concentration index in HRQOL among the employed and unemployed before matching. **Table F.** Horizontal inequity of EQ-5D scores for the employed and unemployed before matching.

## Data Availability

These data were drawn from the fifth Chinese National Health Services Survey of Shaanxi Province, which is not open to everyone. Researchers who want to use the data should contact Zhongliang Zhou (zzliang1981@163.com).

## Abbreviations

HRQOL: Health-related quality of life
EQ-5D: EuroQol 5 dimensions
CI: Concentration index
HI: Horizontal inequity
