# Supplementary material for: Comparison of inequity in health-related quality of life among unemployed and employed individuals in China
Source: BMC Public Health. 2021 Jan 6;21:52. doi: 10.1186/s12889-020-10038-3 (PMC7789144; doi:10.1186/s12889-020-10038-3)
Supplement: Supplementary file 1 — Additional file 1: Table A. Chinese time trade-off utility values for EQ-5D health states. Table B. The multivariate imbalance measure L1 before and after coarsened exact matching. Table C. Distribution of the three response alternatives for EQ-5D in each dimension for the employed and unemployed before matching. Table D. The values for EQ-5D utility and each dimension for the employed and unemployed before matching. Table E. Decomposition of the concentration index in HRQOL among the employed and unemployed before matching. Table F. Horizontal inequity of EQ-5D scores for the employed and unemployed before matching. [file 12889_2020_10038_MOESM1_ESM.docx]

**Table A** Chinese time trade-off utility values for EQ-5D health states

| **C** | **MO2** | **MO3** | **SC2** | **SC3** | **UA2** | **UA3** | **PD2** | **PD3** | **AD2** | **AD3** | **N3** |
| --- | --- | --- | --- | --- | --- | --- | --- | --- | --- | --- | --- |
| 0.039 | 0.099 | 0.246 | 0.105 | 0.208 | 0.074 | 0.193 | 0.092 | 0.236 | 0.086 | 0.205 | 0.022 |

C is a constant term. The value of MO2, SC2, UA2, PD2 and AD2 represent that the response levels are 2 (i.e., some problems) in the dimensions of mobility, self-care, usual activities, pain/discomfort, anxiety/depression. If the response levels in the above dimensions are 1 (i.e., no problems), the values are 0. MO3, SC3, UA3, PD3 and AD3 in the above dimensions indicate that the response levels are 3 (i.e., extreme problems), 0 if otherwise. The value of N3 means that there are response levels with extreme problems in the five dimensions. For example, the utility score for "32231" was U=1-(0.039+0.246+0.105+0.074+ 0.236+0+0.022) =0.278.

**Table B** The multivariate imbalance measure L_1_ before and after coarsened exact matching

| Variables | Before matching  L_1_ (mean) | After matching  L_1_ (mean) |
| --- | --- | --- |
| Gender | 0.059 (-0.059) | 2.80*10^-15^ (6.70*10^-1^) |
| Age | 0.131 (-0.234) | 5.20*10^-15^ (-4.40*10^-14^) |
| Marital status | 0.053 (-0.103) | 4.30*10^-15^ (2.00*10^-14^) |
| Education status | 0.235 (0.219) | 5.50*10^-15^ (4.90*10^-15^) |
| Economic status | 0.094 (-0.281) | 8.50*10^-15^ (-4.50*10^-14^) |
| Multivariate L_1_ | 0.448 | 6.17*10^-15^ |
| N | 10,622 | 7,857 |

Note: L_1_ reports the L_1j_ measure, which is L_1_ computed for the jth variable separated. The mean is labeled in parentheses reports the difference in means.

**Table C** Distribution of the three response alternatives for EQ-5D in each dimension for the employed and unemployed before matching

| EQ-5D dimensions | Employed (%) | | |  | Unemployed (%) | | | χ^2^ | *p*-value |
| --- | --- | --- | --- | --- | --- | --- | --- | --- | --- |
|  | No problem | Some problems | Extreme problems |  | No problem | Some problems | Extreme problems |  |  |
| Mobility | 97.91 | 1.99 | 0.11 |  | 93.33 | 5.96 | 0.70 | 29.608 | <0.0001 |
| Self-care | 99.19 | 0.74 | 0.08 |  | 95.79 | 3.51 | 0.70 | 38.101 | <0.0001 |
| Activity | 98.56 | 1.27 | 0.17 |  | 94.04 | 4.91 | 1.05 | 37.975 | <0.0001 |
| Pain | 93.77 | 5.97 | 0.26 |  | 86.67 | 12.63 | 0.70 | 21.017 | <0.0001 |
| Anxiety | 95.45 | 4.27 | 0.28 |  | 90.18 | 9.12 | 0.70 | 19.806 | <0.0001 |

**Table D** The values for EQ-5D utility and each dimension for the employed and unemployed before matching

| EQ-5D dimensions | Employed (N=10,337) | |  | Unemployed (N=285) | | *p*-value |
| --- | --- | --- | --- | --- | --- | --- |
|  | Mean | S.D. |  | Mean | S.D. |  |
| Mobility | -0.0022 | 0.0159 |  | -0.0076 | 0.0308 | <0.0001 |
| Self-care | -0.0009 | 0.0107 |  | -0.0051 | 0.0258 | <0.0001 |
| Activity | -0.0013 | 0.0115 |  | -0.0057 | 0.0251 | <0.0001 |
| Pain | -0.0061 | 0.0248 |  | -0.0133 | 0.0359 | <0.0001 |
| Anxiety | -0.0042 | 0.0204 |  | -0.0093 | 0.0298 | <0.0001 |
| EQ-5D | 0.9815 | 0.0716 |  | 0.9513 | 0.1354 | <0.0001 |

**Table E** Decomposition of the concentration index in HRQOL among the employed and unemployed before matching

| Variables | Employed |  |  |  | Unemployed | | |
| --- | --- | --- | --- | --- | --- | --- | --- |
|  | dy/dx | Contribution | % |  | dy/dx | Contribution | % |
| Female (Ref: Male) | 0.0004 | 0 | -0.1017 |  | 0.0403* | 0.0010 | 11.3792 |
| 30–44 (Ref: 15-29) | -0.0052** | -0.0002 | -5.7004 |  | -0.0205 | -0.0003 | -2.8663 |
| >45 | -0.0222*** | 0.0008 | 26.9478 |  | -0.0336 | 0.0007 | 7.6621 |
| Marriage (Ref: Single) | -0.0011 | 0 | -0.1572 |  | -0.0146 | -0.0001 | -1.2667 |
| Widowed and divorced | -0.0153*** | 0.0001 | 1.0316 |  | -0.0820 | 0.0005 | 5.2598 |
| Middle school (Ref: Elementary school and below) | 0.0218*** | -0.0012 | -39.4362 |  | 0.1087*** | -0.0054 | -60.3091 |
| Senior high school | 0.0235*** | 0.0006 | 18.9608 |  | 0.1113*** | 0.0027 | 29.7851 |
| College degree and above | 0.0238*** | 0.0019 | 62.9044 |  | 0.0756** | 0.0019 | 21.4971 |
| Basic health insurance (Ref: No health insurance) | 0.0007 | -0.0001 | -2.5238 |  | 0.0145 | -0.0008 | -8.7841 |
| Commercial insurance and other insurance | 0.0022 | 0.0002 | 6.9964 |  | 0.0133 | 0.0006 | 6.9210 |
| Non-daily smoking (Ref: No smoking) | -0.0030 | 0 | 0.0762 |  | 0.0148 | 0 | -0.4867 |
| Smoking daily | -0.0016 | 0 | -0.0362 |  | 0.0577** | 0.0008 | 9.4750 |
| Drinking alcohol (Ref: No drinking) | 0.0050*** | 0.0001 | 4.8125 |  | 0.0236 | 0.0004 | 4.8248 |
| Lower group (Ref: Lowest group) | 0.0034 | -0.0003 | -9.4286 |  | 0.0439 | -0.0037 | -41.8065 |
| Medium group | 0.0053** | 0 | -0.0047 |  | 0.0418 | 0 | -0.2442 |
| Higher group | 0.0046** | 0.0004 | 12.7183 |  | 0.0253 | 0.0022 | 24.1975 |
| Highest group | 0.0051** | 0.0008 | 28.0807 |  | 0.0371 | 0.0063 | 70.3744 |
| CI | 0.0030 |  |  |  | 0.0089 |  |  |

**p*< 0.1, ***p*< 0.05, ****p*< 0.01.

**Table F** Horizontal inequity of EQ-5D scores for the employed and unemployed before matching

|  | Employed | Unemployed |
| --- | --- | --- |
| Contribution of need variables (age-gender) | 0.0006 | 0.0014 |
| Contribution of control variables | 0.0025 | 0.0053 |
| Residual | -0.0001 | 0.0022 |
| CI | 0.0030 | 0.0089 |
| HI | 0.0024 | 0.0075 |
